# Harnessing AI for public health: India's roadmap

**DOI:** 10.3389/fpubh.2024.1417568

**Published:** 2024-09-27

**Authors:** Manisha Nitin Gore, David Bamidele Olawade

**Affiliations:** ^1^Faculty of Medical and Health Sciences, Symbiosis Community Outreach Programme and Extension, Symbiosis International (Deemed University), Pune, Maharashtra, India; ^2^Department of Allied and Public Health, School of Health, Sport and Bioscience, University of East London, London, United Kingdom; ^3^Department of Research and Innovation, Medway NHS Foundation Trust, Gillingham, NY, United Kingdom

**Keywords:** India, artificial intelligence, public health, challenges, policies

## Present scenario of AI in public health

Artificial Intelligence (AI), defined as the simulation of human intelligence processes by machines, especially computer systems, has emerged as a transformative force across various sectors, including public health. Currently, the application of AI in Public Health cuts across data visualization and predicting health outcomes, including patterns and likelihood of diseases (1, 2). AI algorithms can now be used to analyze complex data and manage health records (2). Following a study among Spanish-speaking individuals by the National Institutes of Health, an AI chatbot was 90% accurate in predicting the risk of developing type 2 diabetes. The chatbots collected and analyzed patients' data, physical activities, dietary habits, and social media information to arrive at this result (3).

With the use of AI tools, patients can access medical services remotely and obtain information to improve their mental health (3). From a public health perspective, AI helps improve health education and promotion by providing personalized health advice to users according to their preferences, medical history, and lifestyle (4). AI chatbots have been beneficial in healthcare training and virtual simulation in a human-friendly approach (5). Thus, the use of AI in health promotion and disease prevention is noteworthy in disease management and rehabilitation (3).

Largely, AI can facilitate data collection, analyze causes and effects, summarize patient information, reduce health disparities, and promote public health services (3, 6, 7). However, due to the nascency of modern AI technology, AI use may result in unforeseeable circumstances, such as misinterpretation, especially when it is not used effectively or is heavily relied on. Therefore, public health professionals must be discerning and learn to interpret and use AI appropriately (1).

## Public health challenges in India

This paper aims to specifically explore the application of AI in the Indian public health context by considering its unique socio-cultural and economic landscape. India's public health dynamics are shaped by diverse factors, including its vast socio-cultural diversity, demographic variations, economic disparities, and environmental challenges (8). This complexity presents a dual challenge: the persistent burden of communicable diseases alongside the rapid rise of non-communicable diseases (NCDs). Communicable diseases such as respiratory infections, Tuberculosis, Malaria, and HIV/AIDS remain significant public health concerns. At the same time, the prevalence of NCDs, including cardiovascular diseases and diabetes, is on a steep upswing (9). Alarming indicators in maternal and child health, such as high rates of maternal and neonatal mortality and child malnutrition, further underscore the severity of the situation (10).

The rise of non-communicable diseases, particularly diabetes, is of special concern in India, which is known as the 'diabetes capital of the world'. The Indian Council of Medical Research reports a staggering 101 million diagnosed cases of diabetes, further compounded by an additional 136 million individuals classified as pre-diabetic (11). This prevalence emphasizes the urgent need for preventive measures that are tailored to the Indian context. Additionally, the upward trends in hypertension and obesity rates highlight the broader public health challenges facing India (12, 13).

## AI for India's public health scenario

In India, the utilization of artificial intelligence (AI) for addressing public health challenges has emerged as a promising avenue, offering innovative solutions to improve healthcare delivery and outcomes. AI technologies have been particularly instrumental in predictive healthcare analysis and the automatic diagnosis and screening of diseases (14). Predictive healthcare analysis leverages AI algorithms to analyze vast datasets comprising patient demographics, clinical records, environmental factors, and socio-economic indicators to forecast disease trends, identify high-risk populations, and inform proactive intervention strategies (15). By analyzing historical data and identifying patterns, AI can predict disease outbreaks, epidemics, and emerging health threats with greater accuracy and timeliness, enabling public health authorities to allocate resources efficiently and implement targeted preventive measures (2, 16).

For non-communicable diseases like diabetes, AI offers unique advantages. Unlike other NCDs such as smoking-related diseases, diabetes management in India requires a tailored approach that considers the country's dietary habits, physical activity levels, and access to healthcare services. AI can aid in personalized treatment planning by analyzing patient data, including lifestyle, genetic factors, and social determinants of health. For example, AI can predict individual risk factors and offer customized interventions, such as dietary recommendations or reminders for physical activities, which are culturally appropriate and feasible for the Indian population. This personalized approach is crucial in a country where health literacy varies widely and access to healthcare services is uneven.

Furthermore, AI-driven automatic diagnosis and disease screening hold significant promise in enhancing healthcare accessibility and efficiency, especially in resource-constrained settings such as India (17). Machine learning algorithms trained on large datasets of medical images, such as X-rays, MRIs, and CT scans, can assist healthcare providers in diagnosing various diseases, including tuberculosis, cancer, and diabetic retinopathy, with high accuracy and speed (18). These AI-enabled diagnostic tools have the potential to overcome challenges related to the shortage of trained healthcare professionals and the uneven distribution of medical expertise across different regions of India (19).

Moreover, AI technologies are being applied to streamline healthcare workflows, optimize treatment protocols, and personalize patient care. Natural language processing algorithms enable the automated analysis of unstructured clinical notes and medical literature, facilitating evidence-based decision-making and clinical guideline adherence (18). Additionally, AI-driven telemedicine platforms and mobile health applications empower patients to access healthcare services remotely, receive real-time health monitoring, and engage in self-management practices, thereby improving health outcomes and reducing healthcare disparities, particularly in rural and underserved areas (2). With its capabilities to address both preventive and therapeutic aspects of non-communicable diseases (NCDs), AI holds promise in alleviating the substantial burden of morbidity and mortality associated with NCDs in India. Furthermore, it offers multifaceted assistance in NCD management, encompassing disease prediction, patient monitoring, treatment adherence, and follow-up.

In the context of public health promotion, AI can also play a significant role in addressing malnutrition, particularly among vulnerable populations such as children and the older adult (20). Malnutrition remains a pressing issue in India, where undernutrition coexists with rising rates of obesity, particularly in urban areas (21). AI can help identify at-risk populations by analyzing factors such as income levels, access to food, and dietary habits. For instance, AI-powered tools can monitor the growth patterns of children and alert healthcare workers to early signs of malnutrition, enabling timely interventions (22). Similarly, AI can assist in developing personalized nutrition plans for the older adult, considering their specific dietary needs and potential health conditions, thereby improving their overall health outcomes (23).

Spatial modeling emerges as a crucial component of AI application in public health. Utilizing geographic data analysis, spatial modeling identifies health trends and predicts disease spread. Machine learning algorithms analyze extensive data, including satellite imagery, to aid in resource allocation and interventions (2). For instance, these techniques have been instrumental in forecasting the spread of diseases like Dengue fever (24). Infectious diseases exhibit spatial correlations, and spatial modeling enables visualization, clustering, hotspot detection, and risk factor identification, thereby enhancing surveillance and prevention strategies. India's diverse geography and disease distribution patterns offer ample opportunities for the implementation of spatial modeling. These models have proven particularly useful in understanding the spread factors of diseases such as COVID-19 at the district level in India (25).

Additionally, AI can monitor, organize, and visualize disease outbreaks based on geographical location, time, and infectious pathogens, utilizing real-time data from diverse sources such as social media, news reports, and official health reports. For example, “Arogya Setu” was developed by the Indian Government and stakeholders like the National Information Center to offer real-time contact tracing and self-assessment during critical periods like the COVID-19 pandemic. With 210 million downloads, it aided data-driven responses, saving lives (26).

However, despite the promising applications of AI in addressing public health challenges in India, several barriers and limitations exist. These include the need for robust data governance frameworks to ensure data privacy and security, the requirement for skilled workforce development to effectively leverage AI technologies, and the necessity for regulatory oversight to ensure the safe and ethical deployment of AI-driven healthcare solutions (19). Furthermore, addressing issues related to algorithmic bias, interoperability of health information systems, and the digital divide among different population groups is crucial to maximize the impact of AI in public health in India.

Moreover, the strategy of nurturing partnerships between academia, industry, government, and nonprofit organizations, though beneficial, has limitations. For example, the complexity of diabetes management requires not just technological solutions but also lifestyle and behavioral interventions at the micro level (27). This recommendation may not address the immediate, individual-level challenges faced by diabetic patients, such as adherence to treatment plans, diet, and exercise routines. The partnership approach tends to focus more on upstream determinants, which, while essential, may overlook the nuanced, downstream determinants of health that directly impact patients' daily lives.

To overcome these limitations, a more focused approach is necessary, one that integrates AI applications with community-based health strategies. By combining AI's predictive and personalized capabilities with on-the-ground public health interventions tailored to specific communities, India can more effectively address the growing diabetes epidemic. For instance, community health workers can use AI tools to monitor patient adherence to diabetes management plans in real-time, offering immediate support and adjustments as needed (27). This approach not only bridges the gap between high-level AI applications and individual patient needs but also ensures that the solutions are sustainable and culturally appropriate.

Table 1 provides a comprehensive overview of the challenges associated with the use of AI in public health management in India. While these challenges are not unique to India, the table highlights the need for a localized strategy that integrates AI with existing public health infrastructure and cultural practices. Addressing these challenges requires concerted efforts from policymakers, healthcare professionals, researchers, and stakeholders to ensure the responsible, equitable, and effective integration of AI technologies into public health systems.

**Table 1:** Challenges and strategic approaches for AI integration in public health management in India.

**Challenges**	**Description**	**Strategic approaches**
Data accessibility and quality	Limited access to high-quality healthcare data, particularly in rural areas, impedes the development and effectiveness of AI algorithms	Develop a national digital health infrastructure with standardized data collection protocols and invest in expanding digital access in rural areas
Infrastructure and connectivity	Inadequate infrastructure and connectivity in remote regions restrict the implementation of AI-driven healthcare solutions	Enhance digital infrastructure with a focus on last-mile connectivity and incentivize public-private partnerships to improve technology access
Cultural and linguistic diversity	India's diverse cultural and linguistic landscape presents challenges for AI systems in processing local languages and providing culturally sensitive care	Develop AI models that are trained on multilingual datasets and incorporate cultural nuances to ensure broader accessibility and acceptance
Healthcare workforce skills	Shortage of professionals skilled in AI technologies within the healthcare sector limits the adoption and utilization of AI solutions	Implement interdisciplinary training programs for healthcare workers and data scientists, and promote AI literacy through continuous education
Regulatory framework and compliance	Lack of comprehensive regulations governing AI in healthcare raises concerns about data privacy, security, and the ethical use of AI technologies	Establish a robust regulatory framework that addresses data privacy, security, and ethical considerations, including algorithmic transparency and accountability
Financial resources and funding	Limited financial resources allocated for AI research and development pose barriers to scaling up AI-driven public health initiatives	Increase funding for AI in healthcare through government grants, international collaborations, and private sector investments, with a focus on scalable solutions
Equity and access	Ensuring equitable access to AI-driven healthcare solutions across socio-economic and geographical divides remains a challenge, exacerbating healthcare disparities	Design AI applications that are affordable and accessible, with targeted interventions for underprivileged communities, and ensure equitable distribution of resources
Trust and acceptance	Building trust and acceptance among healthcare professionals and the public toward AI technologies requires transparency, education, and evidence-based outcomes	Engage in community outreach and education campaigns to demystify AI technologies, involve local stakeholders in AI deployment, and ensure AI solutions are evidence-based and user-friendly

## Comparison examples from other LMICs and continents

In comparison, other Low- and Middle-Income Countries (LMICs) like Brazil and Kenya have also leveraged AI to improve healthcare, albeit in different ways. In Brazil, AI has been utilized in telemedicine to extend healthcare services to remote areas (28), similar to India's approach. However, Brazil has also focused on AI in mental health, developing chatbots that provide psychological support in underserved communities (29). Kenya, on the other hand, has implemented AI-driven mobile health platforms to combat malaria and maternal health issues (30), demonstrating AI's versatility in addressing diverse health challenges. These examples illustrate that while the challenges of implementing AI in healthcare are global, the solutions must be tailored to the specific needs and contexts of each country.

In the United States, AI applications in healthcare have encountered challenges such as data privacy concerns, interoperability issues, and algorithmic biases (31, 32), similar to those in India. However, the U.S. has also seen successes, particularly in the use of AI for personalized medicine and precision health, where AI is used to tailor healthcare treatments to individual genetic profiles. While this is a promising area, it also underscores the importance of data security and the ethical use of AI. In contrast, China's extensive use of AI in public health has demonstrated success in predictive analysis and disease outbreak management (33). China's AI strategies often involve large-scale data collection and government-led initiatives, which have proven effective in managing public health emergencies, such as the COVID-19 pandemic. However, this approach has also faced scrutiny over data privacy and ethical concerns, highlighting the trade-offs between public health benefits and individual privacy rights.

In Europe, countries like Germany have made strides in integrating AI into their healthcare systems, yet they too grapple with regulatory and standardization challenges (34). Germany's approach emphasizes the importance of patient data protection and the need for robust ethical frameworks, which can serve as a model for other countries looking to balance innovation with privacy concerns. These international examples highlight that while AI offers promising solutions, the associated challenges are global and necessitate tailored approaches to address specific regional and cultural contexts. For India, this means developing AI strategies that not only harness the technology's potential but also respect and integrate the country's diverse cultural and socio-economic landscape.

## Road ahead and way forward for AI applications in public health in India

The journey toward harnessing the full potential of artificial intelligence (AI) in public health in India requires a strategic and collaborative approach, encompassing various stakeholders, innovative solutions, and sustained efforts. Several key considerations and action points emerge to maximize the impact of AI applications in addressing public health challenges in India:

Firstly, establishing robust data infrastructure and governance frameworks is essential. This involves creating interoperable health information systems, ensuring data quality and standardization, and adhering to stringent data privacy regulations. By fostering partnerships for data sharing while safeguarding patient confidentiality, India can unlock the full potential of AI-driven public health initiatives.

Investing in capacity building and workforce development is critical. Equipping healthcare professionals, data scientists, and policymakers with the necessary skills and knowledge is essential for the effective utilization of AI technologies in public health. Through collaborative efforts and interdisciplinary training programs, India can cultivate a skilled workforce adept at harnessing AI for improved healthcare outcomes.

Promoting innovation and fostering collaboration should be guiding principles along this path. Encouraging entrepreneurship, supporting start-ups, and facilitating cross-sector collaborations are pivotal in accelerating the development and deployment of scalable AI-driven healthcare solutions. By nurturing partnerships between academia, industry, government, and non-profit organizations, India can co-create and implement AI-enabled public health programs tailored to its unique context, driving progress toward Health for All.

Establishing a clear regulatory framework and ethical guidelines is imperative to ensure the responsible and equitable utilization of AI in public health. Policymakers must address issues related to data privacy, security, and algorithmic bias through comprehensive legislation and regulatory oversight. Engaging stakeholders in the development of ethical guidelines fosters transparency, trust, and acceptance of AI-driven interventions, paving the way for sustainable impact in India's public health landscape.

## References

[1] Park CW Seo SW Kang N Ko B Choi BW Park CM. Artificial intelligence in health care: current applications and issues. J Korean Med Sci. (2020) 35:e379. 10.3346/jkms.2020.35.e379 33140591 PMC7606883

[2] Olawade DB Wada OJ David-Olawade AC Kunonga E Abaire O Ling J. Using artificial intelligence to improve public health: a narrative review. Front Public Health. (2023) 11:1196397. 10.3389/fpubh.2023.1196397 37954052 PMC10637620

[3] Jungwirth D Haluza D. Artificial intelligence and public health: an exploratory study. Int J Environ Res Public Health. (2023) 20:4541. 10.3390/ijerph20054541 36901550 PMC10002031

[4] Chaix B Guillemassé A Nectoux P Delamon G Brouard B. Vik: a chatbot to support patients with chronic diseases. Health. (2020) 12:804. 10.4236/health.2020.127058

[5] Baglivo F De Angelis L Casigliani V Arzilli G Privitera GP Rizzo C. Exploring the possible use of AI Chatbots in public health education: feasibility study. JMIR Med Educ. (2023) 9:e51421. 10.2196/51421 37910155 PMC10652189

[6] Chubb J Cowling P Reed D. Speeding up to keep up: exploring the use of AI in the research process. AI Soc. (2022) 37:1439–57. 10.1007/s00146-021-01259-0 34667374 PMC8516568

[7] Brynjolfsson E Rock D Syverson C. Artificial intelligence and the modern productivity paradox. In:Agrawal A Gans J Goldfarb A, editors. The Economics of Artificial Intelligence: An Agenda. Chicago, IL: University of Chicago Press (2019), p. 23–57. 10.7208/chicago/9780226613475.003.0001

[8] Narain JP. Public health challenges in India: seizing the opportunities. Indian J Community Med. (2016) 41:85–8. 10.4103/0970-0218.177507 27051080 PMC4799645

[9] GBD 2017 Disease and Injury Incidence and Prevalence Collaborators. Global, regional, and national incidence, prevalence, and years lived with disability for 354 diseases and injuries for 195 countries and territories, 1990-2017: a systematic analysis for the Global Burden of Disease Study 2017. Lancet. (2018). 392:1789–858. 10.1016/S0140-6736(18)32279-7 30496104 PMC6227754

[10] Ministry Ministry of Health and Family Welfare International International Institute of Population Sciences National Family Health Survey-5 2019-2021 India Fact Sheet. Available at: https://dhsprogram.com/pubs/pdf/FR375/FR375.pdf (accessed August 12, 2024).

[11] Anjana RM Unnikrishnan R Deepa M Pradeepa R Tandon N Das AK. Metabolic non-communicable disease health report of India: the ICMR-INDIAB national cross-sectional study (ICMR-INDIAB-17). Lancet Diabetes Endocrinol. (2023) 11:474–89. 10.1016/S2213-8587(23)00119-5 37301218

[12] Varghese JS Venkateshmurthy NS Sudharsanan N Jeemon P Patel SA Thirumurthy H. Hypertension diagnosis, treatment, and control in India. JAMA Netw Open. (2023) 6:e2339098. 10.1001/jamanetworkopen.2023.39098 37870834 PMC10594142

[13] Gupta RD Tamanna N Siddika N Haider SS Apu EH Haider MR. Obesity and abdominal obesity in Indian population: findings from a nationally representative study of 698,286 participants. Epidemiologia. (2023) 4:163–72. 10.3390/epidemiologia4020017 37218876 PMC10204471

[14] Rodriguez RV Sinha S Tripathi S. Impact of artificial intelligence on the health protection scheme in India. Public Adm Policy. (2020) 23:273–81. 10.1108/PAP-03-2020-0019

[15] Ranasinghe HD. Strategic utilization of big data and analytics for enhancing public health responses to the COVID-19 pandemic. Emerg Trends Mach Intell Big Data. (2023) 15:1–5. Available online at: https://orientreview.com/index.php/etmibd-journal/article/view/24/24

[16] Zeng D Cao Z Neill DB. Artificial intelligence–enabled public health surveillance—from local detection to global epidemic monitoring and control. In:Xing L Giger ML and Min JK, editors. Artificial Intelligence in Medicine. Cambridge, MA: Academic Press (2021), p. 437–53. 10.1016/B978-0-12-821259-2.00022-3

[17] Parsa AD Hakkim S Vinnakota D Mahmud I Bulsari S Dehghani L. Artificial Intelligence for Global Healthcare. In:Chatterjee JM Saxena SK, editors. Artificial Intelligence in Medical Virology. Singapore: Springer Nature Singapore (2023), p. 1–21. 10.1007/978-981-99-0369-6_1

[18] Ahmed ST Kadhem SM. Using machine learning via deep learning algorithms to diagnose the lung disease based on chest imaging: a survey. Int J Interact Mobile Technol. (2021) 15:24191. 10.3991/ijim.v15i16.24191

[19] Das SK Dasgupta RK Roy SD Shil D. AI in Indian healthcare: from roadmap to reality. Intell Pharm. (2024) 2:329–34. 10.1016/j.ipha.2024.02.005

[20] Vichave B Jain N Garad P Gandhi N Meshram P. Malnutrition detection using AI. Int J Adv Res Sci Commun Technol. (2023) 3. 10.48175/ijarsct-9692

[21] Nguyen PH Scott S Headey D Singh N Tran LM Menon P. The double burden of malnutrition in India: trends and inequalities (2006–2016). PLoS ONE. (2021) 16:e0247856. 10.1371/journal.pone.0247856 33630964 PMC7906302

[22] Kustiawan TC Nadhiroh SR Ramli R Butryee C. Use of mobile app to monitoring growth outcome of children: a systematic literature review. Digit Health. (2022) 8:20552076221138641. 10.1177/20552076221138641 36386243 PMC9663617

[23] Cioara T Anghel I Salomie I Barakat L Miles S Reidlinger D. Expert system for nutrition care process of older adults. Future Gener Comput Syst. (2018) 80:368–83. 10.1016/j.future.2017.05.037

[24] Hoyos W Aguilar J Toro M. Dengue models based on machine learning techniques: a systematic literature review. Artif Intell Med. (2021) 119:102157. 10.1016/j.artmed.2021.102157 34531010

[25] Dutta I Basu T Das A. Spatial analysis of COVID-19 incidence and its determinants using spatial modeling: a study on India. Environ Chall. (2021) 4:100096. 10.1016/j.envc.2021.100096 38620946 PMC8035805

[26] Anjaria P Asediya V Bhavsar P Pathak A Desai D Patil V. Artificial intelligence in public health: revolutionizing epidemiological surveillance for pandemic preparedness and equitable vaccine access. Vaccines. (2023) 11:1154. 10.3390/vaccines11071154 37514970 PMC10383160

[27] Kokori E Olatunji G Aderinto N Muogbo I Ogieuhi IJ Isarinade D. The role of machine learning algorithms in detection of gestational diabetes; a narrative review of current evidence. Clin Diabetes Endocrinol. (2024) 10:18. 10.1186/s40842-024-00176-7 38915129 PMC11197257

[28] Morales HM Guedes M Silva JS Massuda A. COVID-19 in Brazil—preliminary analysis of response supported by artificial intelligence in municipalities. Front Digit Health. (2021) 3:648585. 10.3389/fdgth.2021.648585 34713121 PMC8521842

[29] Daley K Hungerbuehler I Cavanagh K Claro HG Swinton PA Kapps M. Preliminary evaluation of the engagement and effectiveness of a mental health chatbot. Front Digit Health. (2020) 2:576361. 10.3389/fdgth.2020.576361 34713049 PMC8521874

[30] Lacey H Jain N Sugimoto M Shimato M Reine I Oria K. Combating malaria in Kenya through collaborative population health education: a systematic review and pilot case study. Infect Dis. (2023) 55:664–83. 10.1080/23744235.2023.2231082 37424519

[31] Olawade DB David-Olawade AC Wada OZ Asaolu AJ Adereni T Ling J. Artificial intelligence in healthcare delivery: prospects and pitfalls. J Med Surg Public Health. (2024) 100108. 10.1016/j.glmedi.2024.100108

[32] Olawade DB Aderinto N Olatunji G Kokori E David-Olawade AC Hadi M. Advancements and applications of artificial intelligence in cardiology: current trends and future prospects. J Med Surg Public Health. (2024) 100109. 10.1016/j.glmedi.2024.100109

[33] Dong J Wu H Zhou D Li K Zhang Y Ji H. Application of big data and artificial intelligence in COVID-19 prevention, diagnosis, treatment and management decisions in China. J Med Syst. (2021) 45:84. 10.1007/s10916-021-01757-0 34302549 PMC8308073

[34] Horgan D Romao M Morré SA Kalra D. Artificial intelligence: power for civilisation–and for better healthcare. Public Health Genomics. (2020) 22:145–61. 10.1159/000504785 31838476

